# Assessing the Implementation of an LGBTQ+ Mental Health Services Training Program to Determine Feasibility and Acceptability During the COVID-19 Pandemic

**DOI:** 10.1007/s11121-023-01505-5

**Published:** 2023-03-10

**Authors:** Jessica N. Fish, Evelyn C. King-Marshall, Rodman E. Turpin, Elizabeth M. Aparicio, Bradley O. Boekeloo

**Affiliations:** 1 Department of Family Science, University of Maryland, College Park, 1142 Valley Drive, College Park, MD 20742 USA; 2 University of Maryland Prevention Research Center, College Park, USA; 3 Department of Epidemiology and Biostatistics, University of Maryland, College Park, USA; 4 Department of Behavioral and Community Health, University of Maryland, College Park, USA; 5 Department of Global and Community Health, George Mason University, Fairfax, USA

**Keywords:** LGBTQ+, Sexual and gender minority, Mental health, Mental health services

## Abstract

Despite significant social and legal progress, LGBTQ+ (lesbian, gay, bisexual, transgender, and other sexual and gender minority) populations continue to experience higher rates of mental health and substance use disorders than their heterosexual and cisgender counterparts. Effective LGBTQ+ affirmative mental health care is essential for addressing these disparities but is often limited and difficult to access. The shortage of LGBTQ+ affirmative mental health care providers results from the absence of required and accessible LGBTQ+-focused training and technical assistance opportunities for mental health care professionals. This study evaluates the implementation of our COVID-19 adapted, completely virtual, organization- and therapist-focused training program to improve the mental health workforce’s cultural competence in working with the LGBTQ+ community: the *Sexual and Gender Diversity Learning Community* (SGDLC). Guided by an expanded RE-AIM model, we used administrator and therapist feedback to assess SGDLC implementation factors to understand how it may be best translated for scaled-up promotion and widespread adoption. Assessment of the initial reach, adoption, and implementation of the SGDLC indicated that it had strong feasibility; reports on satisfaction and relevance support the SGDLC’s acceptability. Maintenance could not be fully assessed from the short study follow-up period. Still, administrators and therapists expressed an intent to continue their newfound practices, a desire for continued training and technical assistance in this area, but also concerns about finding additional opportunities for this education.

## Introduction

Lesbian, gay, bisexual, transgender, and other sexual and gender minority (LGBTQ+) populations experience disproportionately high rates of depression, anxiety, suicidality, and substance use disorders compared to their heterosexual and cisgender peers (Ploderl & Tremblay, 2015; Stanton et al., 2021). These disparities stem from experiences of sexuality- and gender-based stigma and discrimination (Argyriou et al., 2021; Hendricks & Testa, 2012; Meyer, 2003). Effective LGBTQ+-affirmative mental and behavioral health care is one strategy for addressing LGBTQ+-related disparities in mental and substance use disorders, although LGBTQ+-affirmative care and be challenging to access (Williams & Fish, 2020; Williams et al., 2021). Many mental health therapists report a lack of confidence and skill in providing affirming and effective care for LGBTQ+ persons (Fish et al., 2022; Rees et al., 2021; Rock et al., 2010; Shelton & Delgado-Romero, 2013). LGBTQ+ persons also report that LGBTQ+ -affirming mental health services—services that acknowledge and integrate the unique experiences of LGBTQ+ people—are difficult to find (Martos et al., 2018; Shelton & Delgado-Romero, 2013). The lack of therapist training in LGBTQ+ competent care, defined as respectful of and responsive to the health beliefs, practices, and needs of diverse clients and communities (Hays, 2001; Sue et al., 2009), is widely attributed to a lack of accessible and effective training opportunities in professional clinical programs and continuing education (Graham et al., 2012; Nowaskie, 2020; Rock et al., 2010). This study introduces and evaluates the implementation of our COVID-19 adapted strategy to improve the mental health workforce’s competence in working with the LGBTQ+ community: the *Sexual and Gender Diversity Learning Community* (SGDLC). Our multifaceted, entirely virtual training program uniquely includes both organizational and therapist change strategies within mental health services. As part of our randomized control trial (RCT) evaluation of the SGDLC and guided by the RE-AIM model (Glasgow et al., 1999, 2019) and its newly expanded constructs (Reilly et al., 2020), we examined factors related to its implementation to understand better how the program may be translated for scaled-up promotion and widespread adoption by mental health therapists and organizations.

### LGBTQ+ Experiences with Mental Health Services

LGBTQ+ populations consistently show elevated risk for poor mental health and substance use relative to their heterosexual, cisgender counterparts (Ploderl & Tremblay, 2015; Stanton et al., 2021). One way LGBTQ+ population health disparities emerge and are maintained is through inadequate health care systems and approaches to care. Many LGBTQ+ people experience barriers to accessing health care (Fish et al., 2021; Williams et al., 2021), particularly in accessing care that acknowledges and is sensitive to the unique experiences that arise from holding a marginalized sexual orientation and/or gender identity/expression. For example, a recent national study of mental and behavioral health centers found that only 17.6% of state-approved substance abuse facilities and 12.6% of state-approved mental health care facilities report providing LGBT-specific services (Williams & Fish, 2020).

Research suggests that LGBTQ+ people are more likely to engage with mental and behavioral health care services than their heterosexual and cisgender peers (Cochran et al., 2017; Dunbar et al., 2017; Platt et al., 2018). However, many LGBTQ+ clients report low levels of satisfaction with their care and experiences of stigma and microaggression (Benjamin et al., 2021; Benson, 2013; Rees et al., 2021; Shelton & Delgado-Romero, 2013; White & Fontenot, 2019). Conversely, LGBTQ+ clients who engage in services that explicitly attend to their unique experiences tend to be more satisfied and report greater persistence in care (Benson, 2013; Senreich, 2009, 2010; White & Fontenot, 2019). In addition, other studies find that LGBTQ+ clients who engage in LGBTQ+ affirmative treatment report positive experiences in therapy and reductions in stress disorder, depression, panic attacks, and social anxiety disorders (Kaysen et al., 2005; Pachankis et al., 2015, 2022; Ross et al., 2007).

Notably, concerns about a lack of LGBTQ+ competence among therapists are not just reported by clients; therapists also acknowledge being un(der)prepared to work with the LGBTQ+ population (Fish et al., 2022; Graham et al., 2012; Rock et al., 2010). This lack of therapist competence can often perpetuate stigma and bias through inaccurate language and wrongful assumptions about LGBTQ+ populations (Rees et al., 2021; Rock et al., 2010; Shelton & Delgado-Romero, 2013). Unfortunately, the lack of LGBTQ+ -focused training and continuing education opportunities for mental health providers remain pressing issues and perpetuate a lack of workforce readiness to adequately serve LGBTQ+ clients (Graham et al., 2012; Rock et al., 2010). This lack of training opportunities, coupled with the growing proportion of people who identify as LGBTQ+ in the United States (Jones, 2022), underscores a mounting gap in care and an urgent need to develop and disseminate training for the mental health workforce to better serve this population.

### The Sexual and Gender Diversity Learning Community Training Program

**Fig. 1:**
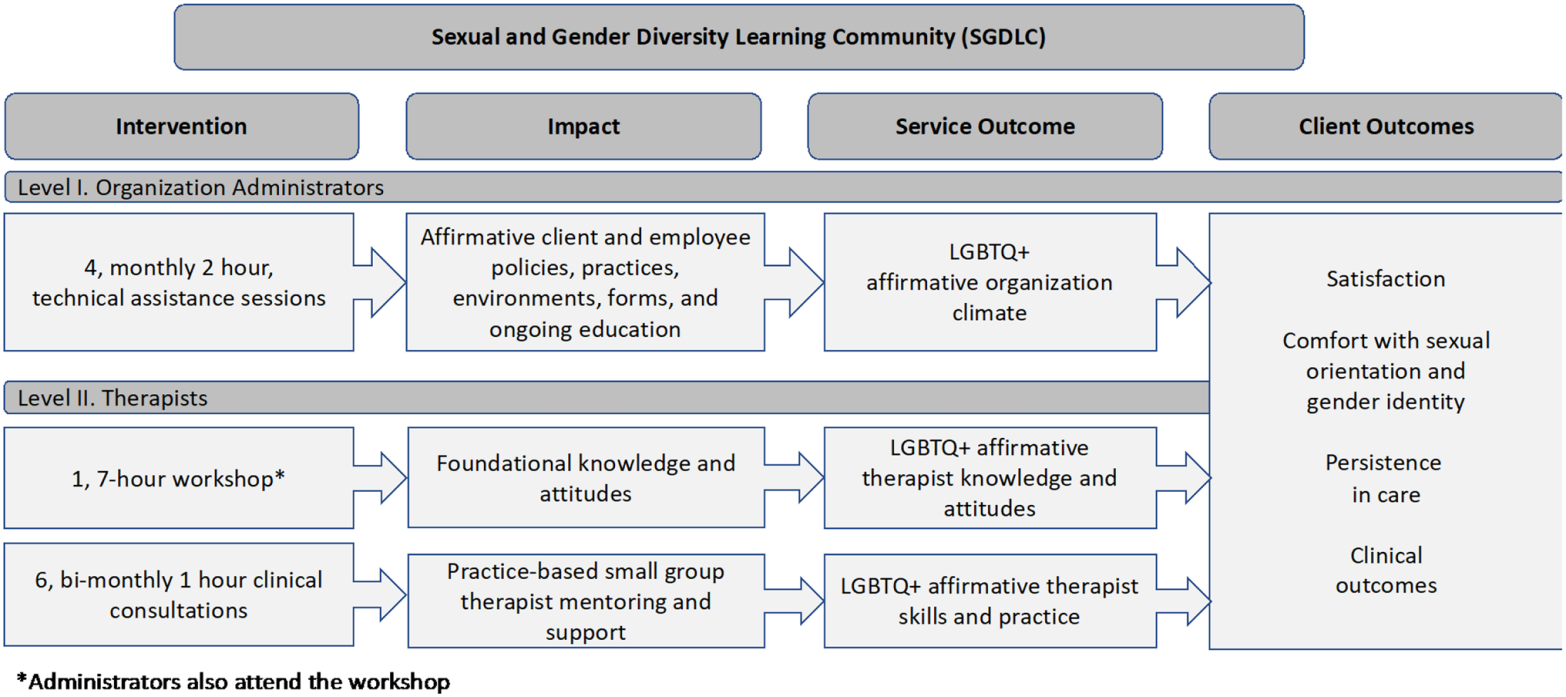
The Sexual and Gender Diversity Learning Community (SGDLC) training model

An earlier iterations of the *Sexual and Gender Diversity Learning Community* (SGDLC; see Fig. 1) was initially developed by Michael Vigorito, LMFT, and Sean Lare, LCSW-C, therapists with expertise in sexual heath, sexual orientation, and gender identity, and then revisioned and revised with the assistance of researchers at the University of Maryland Prevention Research Center at the University of Maryland School of Public Health. The training program evolved through several development, delivery, and refinement cycles (Fish et al., 2022). The SGDLC provides an empirically informed adult learning environment through a 7-hour interactive workshop followed by six hour-long clinical consultation sessions for therapists. We chose this structure because workshops combined with clinical consultations increase therapist competence and improve client outcomes (Frank et al., 2020). In the SGDLC, therapists learn through clinician experts who share their knowledge and experience; discussion of language, concepts, and clinical approaches; activities that explicate values and attitudes; and the practice and integration of new therapeutic skills. Among its most unique features, the SGDLC was designed for multi-level change to improve therapists’ and mental health services organizations’ approach to services for LGBTQ+ clients. Hence, it also includes four 2-hour technical assistance sessions for organizational administrators (e.g., directors, clinical supervisors, human resources [H.R.], and administrators) to receive guidance for assessing their policies regarding data collection forms, electronic medical records (EMR) systems, billing language, bathroom signage, access to inclusive education materials, reception sensitivity, and other systems-level processes that can facilitate or undermine LGBTQ+ client comfort and confidence when engaging with services. Initially, all SGDLC components were designed for in-person, face-to-face learning venues. However, given the onset of the COVID-19 pandemic and the required physical distancing, all SGDLC components were revised for virtual delivery, which became the sole delivery method for implementation. This evolution to virtual rather than in-person on-site training allowed us to provide the SGDLC workshop and clinical consultation sessions to more than one organization at a time and across a broader geographic area.

The novelty of the SGDLC training program and the reliance on an entirely virtual provision of all SGDLC training and technical assistance components made the implementation of the virtual SGDLC program uncharted territory. As a result, we were immediately confronted with many implementation questions in the delivery and adoption of the virtual SGDLC during the early months of COVID, including: Is the virtual SGDLC program acceptable and feasible for mental health organization administrators and therapists?

### The Current Study

The RE-AIM framework (Glasgow et al., 1999, 2019), with its recently expanded constructs (see Reilly et al., 2020), guided an examination of the feasibility and acceptability of the SGDLC training program; the effectiveness of the SGDLC was tested in a randomized controlled trial (RCT) reported elsewhere (Boekeloo et al., under review). The RE-AIM framework provides a socio-ecological perspective and thus requires consideration of how multiple levels of change are necessary to bring about the desired outcome, including, for example, organizational- and therapist-level changes. *Reach* often refers to rates of participation and the characteristics of those participating (e.g., representativeness) in the training program. *Effectiveness* reflects an assessment of both the positive and negative outcomes of the training program as experienced by participants or end-users. *Adoption* refers to the number and characteristics of organizations that initiate a program. *Implementation* references the fidelity and challenges of implementing the training program. Lastly, *maintenance* refers to how and to what extent the behavior promoted by the training program becomes routine or institutionalized over the long term.

Although RE-AIM does not explicitly address the constructs of feasibility and acceptability, recent theorizing around the intersections between RE-AIM and the Implementation Outcomes Framework (IOF) reorients these implementation outcomes as “theoretical antecedents” of RE-AIM indicators reach, adoption, implementation, and maintenance (Reilly et al., 2020). In contrast to the RE-AIM model, which focuses on planning and evaluation, particularly during the dissemination of programs, the IOF assesses the process of implementation, including outcomes such as feasibility, appropriateness, and acceptability, among others. Within the IOF, acceptability reflects the perceptions of the program as adequate or satisfactory, and feasibility addresses how successfully the program was delivered within a given context (Proctor et al., 2011; Rabin & Brownson, 2018; Reilly et al., 2020). In the expanded constructs of the RE-AIM framework (Reilley et al., 2020), feasibility and acceptability are formative assessments for broader RE-AIM constructs to be more thoroughly examined in broader dissemination and implementation (Reilly et al., 2020).

**Fig. 2:**
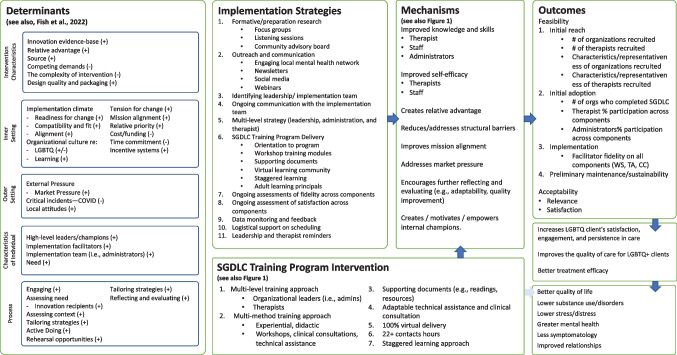
Implementation Research Logic Model (IRLM; Smith, Li, & Rafferty, 2020) for SGDLC implementation and evaluation

Extending our previous work that identified barriers to facilitators of the SGDLC implementation (see Fig. 2), this study aims to capture what was learned about the feasibility and acceptability of the virtual SGDLC when implementing the program as part of an RCT to test the program’s effectiveness with community mental health organizations and therapists (Boekeloo et al., under review). We examine feasibility through assessments of initial: (1) *Reach* based on the number and characteristics of (a) organizations and (b) therapists who respond to invitations to participate in the SGDLC; (2) *Adoption* based on the number of organizations that completed the training program versus those that started but did not complete the training program, as well as administrator and therapist level of participation in each of the SGDLC training program activities; (3) *Implementation* based on the fidelity with which the training program was delivered to both (a) organization administrators and (b) therapists; and (4) *Maintenance* based on (a) organization administrator and (b) therapist qualitative post-test feedback about the intent of participants to continue to implement practices and seek additional training and technical assistance given the ever-evolving language, science, and social/cultural context of LGBTQ+ life in the USA. We further assessed acceptability via participants’ perceptions of relevance and satisfaction with the SGDLC content and delivery based on (a) organization administrator satisfaction with the technical assistance and (b) therapist satisfaction with the workshop and clinical consultations. We also discuss the implications of conducting the training program during the COVID-19 pandemic for future dissemination and training.

## Methods

### Procedures

This study is based on data from a randomized controlled trial (RCT). In each of the two cohorts of 4 organizations (*n* = 8 organizations total), organizations were randomly assigned to the SGDLC training program (intervention) (*n* = 4) and control (*n* = 4) group. Each cohort was then enrolled in a 4-month training cycle. This report focuses on the implementation of the SGDLC training program in the intervention group.

#### Recruitment

For recruitment, the study team compiled a list of mental and behavioral health facilities throughout Maryland via established service and provider networks and listings of mental health services on Maryland Department of Health websites. Recruitment materials were emailed to organizational directors, state regional mental and behavioral health authorities, and researcher personal network contacts in January 2021; contacts were asked to share the recruitment materials throughout their networks.

Interested organizations were instructed to have a CEO/Director, H.R. administrator, or clinical supervisor complete an eligibility survey, which collected information regarding the eligibility characteristics of the organization (e.g., size, services, location) and demographic information about their clientele (e.g., age, race, ethnicity, Medicaid recipients). The survey took an average of 41 min to complete. The research team staff reviewed these initial eligibility survey responses to compile a list of eligible organizations.

#### Eligibility and Enrollment

Eligibility for the program was assessed in three phases. Our first phase was at the organizational level—eligible mental health organizations were required to (1) have two lead administrators willing to serve as point persons for the study (henceforth referred to as “administrators”); (2) have a minimum of five mental health providers (henceforth referred to as “therapists”) eligible and willing to participate in the program; (3) be able to make autonomous decisions regarding the organization’s policies and procedures (e.g., paperwork changes); and (4) not be explicitly branded as an LGBTQ+ focused, substance use rehabilitation, or faith-based services organization. Once organizations were deemed eligible, research staff scheduled an “orientation” meeting with administrators to confirm their eligibility, review the study components and timeline, and discuss the next steps. Following the orientation, administrators solicited interest in the study from a targeted six eligible therapists—five being the minimum and seven being the maximum to remain eligible—from their organization to participate in the study by sharing a therapist orientation video and an FAQ page. Administrators were also required to complete an organization-level online baseline comprehensive assessment of their LGBTQ+ climate and a participant contact form.

The second phase of organizational eligibility focused on therapist eligibility. Once recruited by administrators, therapists were asked to complete a baseline self-assessment of their demographics, client demographics, and their LGBTQ+ competence, which allowed researchers to assess eligibility. Therapists were required to (1) be either provisionally or fully licensed therapists (i.e., clinical social workers, mental health counselors, licensed professional counselors, licensed psychologists, and licensed marriage and family therapists), (2) work at the organization for a minimum of 20 hours a week, and (3) have at least 10 active clients 16 years old or older at the enrolled organization. If administrator and therapist participants appeared eligible, a research team member contacted them to clarify any remaining questions about eligibility.

Organizations took about 3 weeks to complete all enrollment data collection. Once eligibility screening was completed, organizations were officially enrolled in the study and randomized to a study condition (i.e., intervention or control). The intervention and control condition therapists received a list of approximately 15 publicly accessible free online LGBTQ+ clinical competency webinars (https://www.samhsa.gov/lgbtq-plus-behavioral-health-equity?&), whereas the intervention condition therapists also received the SGDLC workshop and SGDLC clinical consultations. The intervention, but not control, condition administrators received the SGDLC workshop and the SGDLC technical assistance. To facilitate engagement in the program components, all study administrators and therapists were given access to an informational website outlining their study condition’s program components, related resources, and timeline of tasks. They were also added to the study condition email list, which provided weekly email updates for administrators and therapists with instructions for upcoming training and data collection activities.

### Intervention Group Training Program

The SGDLC Program consists of three main components: (1) SGDLC workshop for therapists and administrators, (2) SGDLC organizational technical assistance for administrators, and (3) SGDLC clinical consultation for therapists (see Fig. 1). The virtual rather than in-person, on-site provision of the SGDLC allowed the provision of the workshop and clinical consultation sessions to therapists from two organizations combined in each cohort.

#### SGDLC Workshop

The workshop is a 7-hour didactic and interactive seminar delivered virtually at the start of the training program and led by two clinician facilitators who are experts in sexual health, sexual orientation, and gender-identity topics related to mental health and health care. The workshop included four modules: (1) interrogating stereotypes, examining comfort, and understanding the importance of language; (2) LGBTQ+ health disparities and obstacles to care; (3) facilitating sexual health conversations in mental health care; and (4) providing affirmative care and health conversations. The workshop for each cohort was scheduled so that the two intervention organization administrators and therapists could participate together. This was both a cost-efficiency decision and an educational opportunity for cross-organization sharing and learning. The workshop time was largely determined by the administrator and therapist’s choice from options provided by the trainers. Zoom was used as the virtual venue for the workshops. The workshop modules were presented over two consecutive days for cohorts 1 and over one day during cohort 2 due to scheduling conflicts. All participants who completed the 7-hour workshop were offered 7 continuing education units (CEUs) from the National Association of Social Workers (NASW).

#### SGDLC Clinical Consultations

Clinical consultations (CC) for therapists consisted of a series of six 1-hour sessions for intervention condition therapists with one expert clinical trainer. CCs were delivered every 2 to 3 weeks for 3 months. The CC sessions each had a topic for discussion: Collection of sexual orientation and gender identity data; mental health care with lesbian, gay, and bi+ clients; mental health care with transgender and gender non-binary clients; substance use disorder treatment with LGBTQ+ clients; facilitating sexual health conversations with lesbian, gay, and bi+ clients; and facilitating sexual health conversations with transgender and gender non-binary clients. Participants were asked to read an article related to the topic before meeting and bring their clinical challenges regarding LGBTQ+ clients for discussion. The facilitator reviewed case examples when the participants did not have cases to discuss. The facilitator assisted participants in identifying and resolving discomfort related to the topic and provided feedback on improving the clinical skills they demonstrated during the discussion. For each CC, the number of therapists was limited to 12. Each session was offered at two different times so that each therapist from the two participating organizations per study cohort could register to attend the most convenient option.

#### SGDLC Technical Assistance (TA)

Technical assistance (TA) sessions addressed organizational policies and practices related to nine key areas of operation: built environment, human resources, workplace climate, professional development, intake and referral, services and programs, outreach, leadership, and mission and values. Administrators at each intervention organization were provided with four monthly 2-hour sessions of TA by an expert clinical trainer scheduled at a time of mutual agreement. The objective of the TA was to evaluate and change the MHO policies and procedures to improve LGBTQ+ inclusiveness and integrate LGBTQ+ affirmative practices into the system of care. The trainer assessed the MHO policies and procedures during the first and second sessions. The third session focused on setting priority goals for change based on assessing feasibility, opportunity, and importance, as well as developing a plan for change. The fourth session addressed progress toward these goals, identification of challenges and obstacles, and problem-solving. The fourth session also focused on identifying areas with a continued need for change and planning for next steps.

## Sample

Data are from the intervention condition subsample of administrators and therapists only (see Table 1). The sample includes 4 organizations, 8 administrators, and 24 therapists (20 of which completed post-intervention satisfaction surveys).

**Table 1:** Sociodemographic and background characteristics of organizations, therapists, and administrators in the intervention group

Characteristics	Organizations (*n* = 4)	
**Organization structure and function**		
Median number of employees	110	
Median number of therapists	22	
Median annual income	$2,000,000 to $5,000,000	
Provides general outpatient services	100.0%	
Provides intensive outpatient services	50.0%	
Provides school-based mental health services	25.0%	
**Populations served**		
Mean % Non-Hispanic Asian clients	6.8%	
Mean % Non-Hispanic Black clients	46.5%	
Mean % Hispanic/Latino clients	5.0%	
Mean % Non-Hispanic White clients	43.3%	
Mean % clients under 18 years old	38.3%	
Mean % clients 18 to 64 years old	59.7%	
Mean % clients 65 or more years old	2.0%	
Mean % Women clients	64.2%	
Mean % Men clients	34.0%	
Mean % Non-binary clients	1.8%	
Mean % LGBTQ+ clients	20.0%	
Mean % clients with public insurance	78.0%	
Mean % rural	25.0%	
Characteristics	Therapists (*n* = 24)	Administrators (*n* = 8)
**Gender**		
Woman	95.8%	87.5%
Man	4.2%	12.5%
Transgender woman	0%	0%
Transgender man	0%	0%
Non-binary	0%	0%
**Sexual orientation**		
Bisexual	12.5%	0.0%
Gay/lesbian	8.3%	0.0%
Heterosexual	75.0%	100.0%
Other	4.2%	0.0%
**Age**		
18 to 29	20.8%	12.5%
30 to 39	50.0%	50.0%
40 to 59	20.8%	37.5%
60 and older	8.3%	0.0%
**Race/ethnicity**		
Non-Hispanic Asian	0.0%	12.5%
Non-Hispanic Black	25.0%	12.5%
Hispanic/Latino	12.5%	12.5%
Non-Hispanic White	62.5%	50.0%
**Licensure**		
Licensed clinical social worker	50.0%	50.0%
Licensed masters social worker	0.0%	12.5%
Licensed professional counselor	33.3%	12.5%
Licensed mental health counselor	8.3%	0.0%
Licensed graduate professional counselor	8.3%	12.5%
No licensure	0.0%	12.5%
**Length of time at organization**		
Less than 2 years	33.3%	0.0%
2 to 5 years	58.3%	100.0%
6 to 10 years	4.2%	0.0%
More than 10 years	4.2%	0.0%
**Length of practice**		
Less than 1 year	0.0%	-
1 to 3 years	41.7%	-
4 to 10 years	25.0%	-
More than 10 years	33.3%	-

### Measures and Assessments

#### Feasibility

The feasibility of the SGDLC was assessed via initial assessments of reach, adoption, implementation, and maintenance (see Fig. 2).

##### Reach

Reach was assessed through the number and characteristics of the organizations and therapists recruited and engaged in the SGDLC program (see Table 1 for response options to all organizational, administrator, and therapist characteristic measures). *Organization characteristics* were reported by administrators via the online eligibility screening survey, which measured the number of employees and therapists, annual organizational income, and percentages of clients receiving various types of general and intensive outpatient services, as well as school-based mental health services. Also reported were percentages of clients served across race, ethnicity, age, gender, sexual orientation and gender identity, types of insurance, and rurality. *Administrator and therapist characteristics* were self-reported in online surveys measuring gender, sexual orientation, age, race, ethnicity, licensure, length of time at the organization, and length of practice experience.

##### Adoption

Adoption was assessed via the number of organizations that completed the training program versus those that started but did not complete the training program, as well as administrator and therapist level of participation in each of the SGDLC training program components, coded as yes or no based on attendance records.

##### Implementation

We assessed implementation based on the fidelity of all programmatic components, defined as the level of completion of expected programmatic elements by the program facilitators. The facilitators for each activity completed an online fidelity form immediately after each activity was delivered. For the workshop, fidelity was assessed with 42 items across 7 sections (i.e., preparation, orientation, 4 distinct modules, and closing); response options were “Completely Met,” “Partially Met,” and “Not Met.” To summarize many items, we report mean percentages of fidelity items “completely met” for training program workshop activities. For TA, fidelity was measured after each session with items pertaining to 9 elements (e.g., goals of the implementation team were clarified; the needs assessment tool was reviewed); we report percentages of elements “completely met” and “partially met.” For clinical consultations, fidelity was measured at the end of each session with items pertaining to 14 elements (e.g., the facilitator encouraged reflection and discussion to identify biases and discomfort related to sexuality and gender diversity, the facilitator invited participants to comment on one another’s cases), and we report the mean percentages “completely met.”

##### Maintenance

Preliminary maintenance indicators were gleaned from virtual semi-structured interviews via Zoom using questions derived from the Consolidated Framework for Implementation Research framework interview guide (Damschroder et al., 2015). The interviews were conducted separately for each intervention group organization approximately 1 month after study participation termination and included administrators and therapists (*n* = 4 interviews). During the interviews, participants were asked to reflect on the participation experience, as well as the quality and impact of the overall program, and then the positive and negative aspects of each activity. Interviews were professionally transcribed and reviewed by three research team members (EKM, BOB, JNF) for concepts that contextualized and expanded upon the quantitative data assessments. We used content analysis techniques to analyze the qualitative data. Although similar to thematic analysis and sometimes used interchangeably (Vaismoradi & Snelgrove, 2019; Vaismoradi et al., 2013), the goal of content analysis is to describe characteristics of the content of media (i.e., documents, interviews; Bloor & Wood, 2006) and uses a descriptive approach in coding. Specifically, following summative content analysis (Hsieh & Shannon, 2005), we read through the data and reviewed participant responses specific to long-term, follow-up, and next steps of the SGDLC program. Selected quotes were most illustrative of trends found across all focus groups.

#### Acceptability

We examined the extent to which the SGDLC was relevant, palatable, and delivered satisfactorily based on feedback from administrators regarding organizational TA and from therapists regarding the workshop and clinical consultations. This was assessed quantitatively through reported satisfaction scores and qualitatively through open-ended responses in immediate post-test online feedback surveys for each training program component. We measured 8 satisfaction items for both workshops and TA, 7 additional satisfaction items for workshops only, and a separate set of 7 satisfaction items for clinical consultations only (see Tables 2 and 3 for the wording of these items). All items were measured on a 5-point Likert scale, with scores ranging from “strongly disagree” (1) to “strongly agree” (5). Mean scores and standard deviations were calculated for all items.

**Table 2:** Mean satisfaction scores^a^ (and standard deviations) for intervention workshops and technical assistance among therapists (*n* = 20) and administrators (*n* = 8) respectively

	Workshop: therapists	Technical assistance: admin
**Workshop and technical assistance items**		
The objectives of the program were clear	4.67 (0.3)	4.93 (0.1)
The virtual format was adequate	4.73 (0.2)	4.80 (0.1)
I was satisfied with the instructor knowledge and expertise	4.87 (0.1)	4.95 (0.1)
I was satisfied with the instructor teaching ability	4.87 (0.3)	4.95 (0.1)
I was satisfied with the quality of the instruction	4.87 (0.3)	4.90 (0.1)
I was satisfied with the teaching material used during the program	4.60 (0.3)	4.87 (0.1)
The program met my expectations	4.47 (0.5)	4.93 (0.1)
The program improved my knowledge about LGBTQ+ health disparities and affirmative mental health services	4.67 (0.4)	4.93 (0.1)
**Additional workshop items**		
The program improved my comfort and confidence with providing LGBTQ+ affirmative mental health care	4.81 (0.2)	-
The program improved my skill and ability to provide LGBTQ+ affirmative mental health care	4.73 (0.1)	-
I was satisfied with program organization/arrangement	4.47 (0.5)	-
I was satisfied with administrative processes related to accessing the program	4.21 (0.8)	-
I was satisfied with the choice and amount of interaction and activities	4.27 (0.6)	-
I was satisfied with the cost of the program to you for participation	4.43 “(0.7)	-
I was satisfied with the duration of the program	4.47 (0.6)	-

91.7% of therapists and 100.0% of administrators reported they would recommend this program to a friend or colleague

^a^Scores reflect a Likert Score ranging from “strongly disagree” (1) to “strongly agree” (5)

**Table 3:** Mean satisfaction scores^a^ (and standard deviations) with clinical consultations among intervention group therapists (*n* = 20)

The objectives of the program were clear	4.89 (0.1)
I was able to bring up my practice experiences in this session	4.63 (0.2)
I benefited from other clinicians discussing practice experiences in this session	4.82 (0.1)
This session was effective in preparing me to address mental health of LGBTQ+ clients	4.82 (0.1)
I was satisfied with the quality of this session	4.92 (0.1)
The facilitator was well prepared for the clinical consultation session	4.95 (0.1)
The facilitator was receptive to participant comments and questions	4.96 (0.1)

98.1% of therapists reported they would recommend this program to a friend or colleague

^a^Scores reflect a Likert Score ranging from “strongly disagree” (1) to “strongly agree” (5)

## Results

### Feasibility: Reach

Twenty-nine organizations completed our initial eligibility survey; 10 of the 29 organizations were deemed eligible based on our RCT requirements and were invited to the first two cohorts of the study. The most common reasons for organizational exclusion were not having at least 5 therapists with 10 clients each or not being a general mental health services organization (e.g., substance use rehabilitation or faith-based focus). Two eligible organizations ultimately opted not to participate; one was moving office locations, and one was going through reaccreditation—both expressed concern about their ability to comply with the research participation requirements given these competing demands. Enrolled organizations included a median of 110 employees, with a median of 22 therapists. All organizations provided general outpatient services, and half provided intensive outpatient services. Client populations were on average 46.5% Black, 43.3% White, 59.7% 18 to 64 years old, 64% women, 78% utilizing public insurance, and 20% LGBTQ+. Therapists were 95.8% women, 75% heterosexual, 62.5% White, and 25% Black. Administrators were 88.9% women, 100% heterosexual, 66.7% White, and 11.1% Black.

### Feasibility: Implementation

The mean percentages of fidelity targets “completely met” for the workshop elements were 100% for each item except one, which was 98.3% (Table 4). For clinical consultations, 75% of the targets were met entirely. Percentages of fidelity targets completely met for TA were high, though there was some variability across items (Table 5): 78.7% of targets were met entirely, with 96.4% being either wholly or partially met.

**Table 4:** Mean percentages of multi-item fidelity targets completely met for intervention workshops (*n* = 2) and clinical consultations (*n* = 12)^a^ (completed by facilitator)

Workshop: Preparation (3 items)	100.0%
Workshop: Orientation (7 items)	100.0%
Workshop: Interrogating stereotypes, examining comfort, and understanding the importance of language (6 items)	100.0%
Workshop: LGBTQ+ health disparities and obstacles to care (12 items)	98.3%
Workshop: Sexual health conversations in mental health care (6 items)	100.0%
Workshop: Providing affirmative care (4 items)	100.0%
Workshop: Closing (4 items)	100.0%
Clinical consultations (14 items)	75.0%

Percentages partially met and not met are not shown. In some instances, specific items were not applicable for specific sessions, resulting in small differences in denominators

^a^Workshops were delivered to two organizations at a time and fidelity was assessed for each of the four organizations. One-hour clinical consultation sessions were delivered to therapists in two organizations at a time, and fidelity was assessed for each session

**Table 5:** Percentages of fidelity targets met for intervention technical assistance provided to administrators (completed by facilitator)

	Completely met	Partially met
Goals of implementation team were clarified	88.9%	11.1%
The needs assessment tool was reviewed	75.0%	25.0%
The targeted assessment questions on the needs assessment tool were discussed	85.7%	11.1%
The notes from the needs assessment tool were translated into a list of changes for the organization to complete	69.2%	18.7%
An action plan was developed for each item to complete during the TA session	50.0%	42.1%
An action plan was developed for any targeted item not completed during the TA sessions	69.2%	22.2%
Questions about content were clarified	100.0%	0.0%
Next steps were identified	100.0%	0.0%
Clarified any system, staff, or provider-level changes to be implemented	70.5%	29.4%
Mean for all targets	78.7%	17.7%

Percentages not met are not shown. In some instances, specific items were not applicable for specific sessions, resulting in small differences in denominators

Technical assistance sessions were delivered over 4 months across four organizations

### Feasibility: Adoption

Overall, we had high levels of adoption based on our metric of participation. Of the four organizations enrolled in the intervention arm, all four completed the SGDLC training program. Among the administrator participants, all administrators completed all 7 workshop hours, and a mean of 3.5 technical assistance sessions out of 4 were completed per administrator. Among therapists, a mean of 5.5 workshop hours out of 7 were completed, and a mean of 4.1 clinical consultation sessions out of 6 were completed.

### Feasibility: Maintenance

Given that exit interviews with participating organizations and therapists were the last post-test data to be collected and completed 1 month following study termination, we cannot speak to the long-term sustainability of the policies, procedures, and skills adopted by organizations, administrators, and therapists. However, participants spoke at length about changes since completing the training program and hinted at their expectations for and commitment to maintaining what was learned in the SGDLC program.

Administrators and therapists described the application of the training in their everyday activities. A therapist noted that they “got the opportunity to really put it into practice right off the bat.” Another described how they used their new skills when providing services to a young client and their family, “I had a little one that started—she started kind of the coming out process, and I had the suspicion, and she’s young, so—and it really helped going through this and working with the family.” Other participants discussed how the skills they learned improved their approach to working with all of their clients, regardless of gender identity or sexual orientation, “It was helpful to be able to use the interventions and also the—one of the things that sticks out is the intake material. It allowed me to really have a framework that was good for every client, not just for the LGBTQ+ population.”

In addition to the adoption and implementation of therapeutic skills, therapists spoke of their ongoing commitment to updated policies and forms within their organization. “I know we’ve changed intake forms. We’ve changed to have gender identity—we’ve had for sexual orientation”… “I don’t think we’ve changed consent forms, …I think we’re working on referral forms, but maybe there has been a change in the referrals. There’s definitely a change on the intake forms, and there’s a change in the intake process in terms of what people are doing.” Many also discussed next steps and planning for their long-term goals. One administrator stated, “[the program went] very well. We certainly still have more work to do on our end, but I will say, for me, just there is now that running tape in my head, and everything, every policy that I’m looking at, every procedure that we’re considering when we think about building new programs, how are we going to make sure that this is inclusive and affirming, so I think very, very well.” Administrators also spoke to continued efforts to increase LGBTQ+ competency, including seeking out additional training opportunities, “In fact, based on those, I reached out to [local LGBTQ+ organization] about coming in and facilitating a training here for our agency, so very useful.”

### Acceptability

Acceptability was assessed quantitatively through mean satisfaction scores, which were high for both the organizational TA and therapist workshop (see Table 2). Based on our response scale where 4 is “Agree” and 5 is “Strongly Agree,” mean satisfaction scores for all items in both training components were above 4, with the majority of items above 4.8. Mean satisfaction scores for clinical consultations among therapists were also high, with means for all items greater than 4.5, with most items above 4.8 (see Table 3).

Open-ended responses from the satisfaction surveys from administrators and therapists provided additional support for their satisfaction with the program components. Many comments reflected administrators’ comfort and satisfaction with the training facilitators, who were the same throughout the workshops, clinical consultations, and TA. An administrator mentioned that the facilitators during the TA shared “insight and guidance [that was] very valuable; the focus on [the] application of these principles will be beneficial to our agency.” Additional comments mentioned that facilitators were “very patient and talented trainers” and “…[do] an outstanding job of breaking down a large process into small steps…[and] created a safe and non-judgmental atmosphere which allows for honesty and openness.” These comments about safety and comfort were common and emphasized the importance of rapport with facilitators and their ability to be seen as a trusted but also expert source of information and guidance.

When asked to reflect on what specifically benefited participants in the TA, many administrators and therapists reflected on the individualized attention and that the SGDLC addressed each level of the organization, “the hands-on, individualized attention to our organization’s need. I think this was much more helpful” and “learning about the need to change various aspect of the organization to be sensitive to the LGBTQ+ population.” Another administrator mentioned that facilitators really took “time to truly dig into [the organization’s] mission and vision and the hiring/onboarding process. Analyzing and discussing what changes are needed and how to get there as an organization.” When reflecting on the overall program, an administrator mentioned, “the knowledge and suggestions offered through our participation in the SGDLC ha[ve] been invaluable.” Another therapist added that they appreciated the “time and effort that [facilitators] put into teaching us, informing us of best practices, and advising us as to how to put them into practice. Grateful for this opportunity to learn and to improve my clinical skills and to see how to make improvements across our agency for LGBTQ+ affirming and inclusive care.” Ultimately, open-ended comments reiterated administrators’ and therapists’ satisfaction with the program content, components, and delivery, and elaborated on well-received elements of the training program.

## Discussion

Given a dearth of LGBTQ+-focused training for mental health providers, the current study aimed to evaluate the feasibility and acceptability of the Sexual and Gender Diversity Learning Community (SGDLC), a multi-level LGBTQ+ cultural competency training program for mental and behavioral health organizations and therapists that was adapted to virtual delivery because of the COVID-19 pandemic. We used expanded constructs from the RE-AIM framework (Reilly et al., 2020) to guide the assessment of the feasibility and acceptability of the Virtual SGDLC with mental health service organization administrators and therapists. Data strongly suggest that the Virtual SGDLC, all of its components at the organizational and therapist level, is both a feasible and acceptable training program for engaging mental health care organizations and therapists in implementing LGBTQ+-affirmative policies, practices, and skills into their operations and services.

The *reach* of the SGDLC program was adequate in that most organizations with the necessary quota of therapists for study eligibility were enrolled and able to recruit and retain enough therapists to participate. Importantly, these organizations were diverse in size, services, population served, and geographic dispersion within the state; participating therapists were also diverse in terms of age, race, ethnicity, licensure, and length of practice in the field. Although most organizations were ineligible to participate due to our narrowly defined RCT eligibility requirements, it is notable that although only Maryland mental health services organizations were solicited through email and social media communications, 29 organizations expressed interest in participating in training the program. This interest in the SGDLC demonstrates a strong desire for LGBTQ+-focused training and is consistent with limited previous research, which observes therapists’ lack of, but need for, training in this area (Graham et al., 2012; Rock et al., 2010).

The *adoption* of the SGDLC was excellent based on the engagement of administrators and therapists with all of the training program activities. All eight organizations enrolled in the study completed the SGDLC program components. Each of the eight administrators also completed all 4 modules of the 7-hour workshop; all four TA sessions per organization were completed by at least one administrator, with a mean of 3.5 TA sessions completed by each administrator. Each of the 24 therapists enrolled completed a mean of 5.5 workshop modules (out of 7) and 4.1 clinical consultation sessions (out of 6).

The *implementation* of the SGDLC program was, for the most part, excellent across all training program activities, although fidelity was most variable for the clinical consultations and TA sessions. Workshop fidelity was highest, with almost all items reported as completely met by facilitators. The implementation of the clinical consultations and TA components showed a greater degree of variability but still demonstrated strong fidelity, with 75% of clinical consultation components completely met and 96.4% of TA components either completely or partially met. The observed variability in facilitator reports of clinical consultation and TA fidelity is expected since clinical consultations and TA are somewhat malleable training components, given that facilitators need to adapt the training components to meet the therapists’ and organizations’ needs “where they are at.” For example, with clinical consultation, therapists bring active cases for feedback and guidance, which make each session somewhat unique alongside its core objectives. Perhaps even more malleable is TA, which conceptualizes and addresses organizational needs across various issues related to policies, operations, services, EMR, built environment, and paperwork. Although the administrators can directly address many of the issues raised in the TA assessment, many require various organizational leaders—or perhaps even an advisory board’s—approval which can take time and require the education of others in the organization by the SGDLC participant administrators. These experiences were highlighted in the exit interviews, which suggested that administrative and organizational changes were ongoing, even after the program’s termination. Given the inherent idiosyncrasies of these two training components, we believe that the degree to which our facilitators could completely or partially meet these components reflects a strong indicator of feasibility.

Assessments of *maintenance* of the SGDLC program remain preliminary, given that we received feedback from administrators and therapists only 1 month following the termination of the program. Still, narratives in the qualitative exit interviews showed that participants were determined not to revert to old practices. In addition to their intent to continue the practices learned in the SGDLC, they also reported motivation to seek additional training programs to continue building their LGBTQ+ competency. However, they often expressed concerns about when and how they would find new opportunities for this education. The qualitative data also highlighted ongoing activities administrators and therapists engaged in within their organization and practice with clients. For example, administrators discussed ongoing progress with changing policies and practices, including continued efforts to update paperwork and intake procedures. Therapists also discussed how they continued to carefully and sensitively incorporate sexual orientation and gender identity into their therapeutic intake and counseling practices. Again, although preliminary, these findings demonstrate changes in long-standing institutional procedures and codification of changes to maintain LGBTQ+-affirmative practice. Evidence for maintenance was particularly noticeable as administrators described organization-level changes with an intent to continue those practices moving forward. Evidence about whether changes in practice are maintained at the therapeutic level will need to be the focus of future investigations.

Lastly, acceptance was found to be good in that organization administrators highly rated the organizational TA components, and the therapists highly rated the workshop and clinical consultation sessions. Both organizational administrators and therapists described clear advancements in their LGBTQ+ affirmative practices and high satisfaction with all training program activities. The qualitative data from satisfaction surveys and exit interviews further substantiated effectiveness, with participants emphasizing specific qualities of the program, facilitation, and delivery that elevated their consciousness, increased their knowledge, altered their practices, and improved their therapeutic skills when engaging with and providing services for LGBTQ+ clients. Interestingly, much of the qualitative feedback reflects changes in attitudes, knowledge, and skills when working with the LGBTQ+ community, which are well-aligned with the core constructs of culturally competent care (Hays, 2001; Sue et al., 2009). Still, even in the context of their satisfaction with the program, it should be noted that both administrators and therapists routinely described the need for continued training and TA in this area—emphasizing both a heightened conscientiousness when working with LGBTQ+ communities but also the need for more comprehensive LGBTQ+-focused training programs for mental health providers. The forthcoming quantitative findings from the RCT comparing the intervention with the control group effectiveness outcomes will help elucidate the extent to which the SGDLC sensitized administrators and therapists to their gaps in affirmative practices and which gaps remained after the implementation of the SGDLC. Actual quantitative measures of perceived effectiveness in affirmative care may be lower in the SGDLC intervention group, given administrators’ and therapists’ new sensitivity regarding gaps in affirmative practices.

Altogether, our findings suggest that virtually conducting the SGDLC training program during the COVID-19 pandemic was highly feasible and acceptable. One unintended positive consequence of virtual administration was eliminating the need for training and TA staff to travel to provide the training program. This was particularly notable in that the mental health services organizations targeted for the implementation of the SGDLC were widely geographically dispersed across the state. In fact, evidence supporting the virtual administration of SGDLC makes the program suitable and cost-effective for regional and national implementation. That the administrators and therapists rated the program very highly suggests that the virtual delivery did not significantly detract from, and may have enhanced, program feasibility or acceptability. Virtual administration had other potential benefits as well. It allowed workshop and clinical consultation administration to include therapists from more than one organization at a time. Hence, therapists from multiple organizations could learn from each other as they worked to implement their newly acquired knowledge and skills into clinical practice, further enhancing the program’s educational potential. Finally, it necessitated that program implementation materials and communications be organized on a website accessible to all participants both during and after program implementation. Consequently, opportunities to prepare for program implementation, reference materials during program implementation, and engage with materials following program implementation were easily accessible to all program participants at different time points.

It should be noted that although the SGDLC program was administered virtually, all activities were synchronous. This live interaction between participants and trainers is distinct from many of the available LGBTQ+ -focused training opportunities for medical and mental health providers and was likely a critical component to the feasibility and acceptability of the program implementation. Although recorded webinars and static online information materials are informative and can support therapists’ edification on LGBTQ+ topics, the nature of these educational modalities likely limit engagement, retention of information, and long-term behavioral change in clinical practice.

The importance of developing and implementing evidence-based training programs to elevate LGBTQ+-related cultural competence among mental health care providers cannot be overstated. Even with policy and social progress, LGBTQ+ populations continue to demonstrate elevated risk for suicidality, mood disorders, and substance use disorders (Ploderl & Tremblay, 2015; Stanton et al., 2021), and although LGBTQ+ populations are more likely to engage in mental health care, they often to report low satisfaction and quality of care (Cochran et al., 2017; Platt et al., 2018). Findings from this implementation study emphasize the feasibility and acceptability of the virtual SGDLC and its ability to engage and retain mental health organizations and therapists in a comprehensive, multi-level, multi-modal training program designed to elevate the LGBTQ+-affirmative policies and practices of community mental health organization and their therapists—thereby helping to address services gaps for LGBTQ+ affirmative mental health care and substance use disorder treatment (see Williams & Fish, 2020). Importantly, given the necessary changes to accommodate the program launch during the COVID-19 pandemic, our findings show that the SGDLC was feasible and acceptable through full virtual delivery, which primes the SGDLC for national scale-up and implementation.

### Limitations and Future Directions

We have several limitations of note. As with all self-report measures, but particularly in the context of culturally sensitive practice, there is potential for response bias. We also may be limited in our generalizability in that the sample required a narrow definition to conduct our RCT properly; the study organizations all offered general mental and behavioral health care and had at least five therapists with sizeable client loads. Participating organizations are also unique because they voluntarily signed up to receive LGBTQ+ training and technical assistance. Implementation of our program to organizations that may be more resistant to change would likely present unique challenges to program engagement, implementation, and maintenance. As discussed previously, the implementation of our program coincided with the start of the COVID-19 pandemic, which presented unique challenges and opportunities in the delivery of our program. At the same time, these constraints and the impact of the pandemic on mental health professionals cannot be understated (see Fish & Mittal, 2021) and likely impacted the degree to which therapists and administrators were able to engage with the program. That said, our findings suggest that even with these challenges, we observed strong engagement, feasibility, and acceptability of the program, which would likely be less constrained in future iterations.

Beyond our findings supporting the acceptability and feasibility, there were also unique strengths regarding this investigation and the SGDLC program. One strength of this study is that both administrator and therapist perspectives were considered. Other strengths include reviewing both quantitative and qualitative data to provide a more in-depth assessment of specific RE-AIM construct antecedents and that the data were based on the results of two distinct cohorts, which support the replication of the program implementation. The SGDLC program was a multi-level training program addressing the organizational systems and climate, and therapist knowledge, attitudes, and practices. Hence, the organizational changes likely create an environment for therapists to effectively provide affirmative care for LGBTQ+ clients. The virtual administration of the program also provides a unique opportunity to fast-track regional and national scale-up to address the pressing mental health needs of and ongoing service gaps for the LGBTQ+ community in the USA.
