# Correction: Assessing the Implementation of an LGBTQ+ Mental Health Services Training Program to Determine Feasibility and Acceptability During the COVID-19 Pandemic

**DOI:** 10.1007/s11121-023-01634-x

**Published:** 2024-01-08

**Authors:** Jessica N. Fish, Evelyn C. King-Marshall, Rodman E. Turpin, Elizabeth M. Aparicio, Bradley O. Boekeloo

**Affiliations:** 1 Department of Family Science, University of Maryland, College Park, 1142 Valley Drive, College Park, MD 20742 USA; 2 University of Maryland Prevention Research Center, College Park, USA; 3 Department of Epidemiology and Biostatistics, University of Maryland, College Park, USA; 4 Department of Behavioral and Community Health, University of Maryland, College Park, USA; 5 Department of Global and Community Health, George Mason University, Fairfax, USA

**Correction to: Prevention Science** 10.1007/s11121-023-01505-5

The article “Assessing the Implementation of an LGBTQ + Mental Health Services Training Program to Determine Feasibility and Acceptability During the COVID-19 Pandemic”, written by Fish, J.N., King-Marshal, E.C., Turpin, R.E., Aparicio, E.M., and Boekeloo, B.O., was originally published electronically on the publisher’s internet portal on 10 March 2023 without open access. With the author(s)’ decision to opt for Open Choice the copyright of the article changed on 13 November 2023 to © The Author(s) 2023 and the article is forthwith distributed under a Creative Commons Attribution 4.0 International License, which permits use, sharing, adaptation, distribution and reproduction in any medium or format, as long as you give appropriate credit to the original author(s) and the source, provide a link to the Creative Commons licence, and indicate if changes were made. The images or other third party material in this article are included in the article’s Creative Commons licence, unless indicated otherwise in a credit line to the material. If material is not included in the article’s Creative Commons licence and your intended use is not permitted by statutory regulation or exceeds the permitted use, you will need to obtain permission directly from the copyright holder. To view a copy of this licence, visit http://creativecommons.org/licenses/by/4.0/.

The original article has been corrected.

